# Intraoperative radiation therapy for the treatment of recurrent retroperitoneal and pelvic tumors: a single-institution analysis

**DOI:** 10.1186/s13014-018-1168-x

**Published:** 2018-11-20

**Authors:** Tharcisio Machado Coelho, Ricardo César Fogaroli, Antonio Cassio Assis Pellizzon, Douglas Guedes De Castro, Guilherme Rocha Melo Gondim, Maria Leticia Gobo Silva, Michael Jenwei Chen, Henderson Ramos

**Affiliations:** Department of Radiotherapy, A C Camargo Cancer Center, São Paulo, Brazil

**Keywords:** Retroperitoneal and pelvic recurrent tumors, Intraoperative radiation therapy, Local control, Salvage surgery

## Abstract

**Background:**

Patients with recurrent retroperitoneal and pelvic region tumors often require multimodal therapies. Intraoperative radiation therapy (IORT) can deliver high-dose radiation to tumor beds, even if first-line external beam radiation therapy (EBRT) was administered. We evaluated local control (LC) and survival in patients receiving IORT for recurrent tumors.

**Methods:**

We retrospectively analyzed 41 patients with isolated pelvic or retroperitoneal recurrences of colorectal, gynecological, or retroperitoneal primary tumors. Following salvage surgery, all patients underwent tumor bed IORT via electron beam or high dose rate brachytherapy. Isolated IORT (median dose: 15 Gy) was administered to patients who had received first-line EBRT; other patients received IORT (median dose 12 Gy) plus EBRT. Local (LF), regional (RF), and distant failures (DF) were evaluated, and the Kaplan–Meier method and log-rank test were used to evaluate and compare overall survival (OS) from the date of IORT.

**Results:**

Forty-one patients underwent 44 treatments, including 27 (61.3%) isolated IORT and 17 (38.7%) IORT and EBRT combination regimens. The median follow-up was 8.1 years (range: 4.4–11.7 years), and the 2, 5, and 8 year overall LC rates were 87.9, 64.0, and 49.8%, respectively. Regarding resection status, the respective 2, 5, and 8 year LC rates were 90, 76, and 76% for R0 resection and 75, 25, and 0% for R1 resection (*p* < 0.001). The 2, 5, and 8 year OS rates were 68, 43, and 26%, respectively. OS was better among patients with LC (*p* < 0.001). Twenty-four patients (58.5%) experienced a DF, and the 5 year OS rates for the patients with and without DF were 36 and 52%, respectively (*p* = 0.04).

In a multivariate analysis, LF (p = 0,012) and recurrent retroperitoneal sarcoma (p = 0,014) were identified as significant predictors of worse OS. Thirteen patients (31%) developed clinically treatable complications related to IORT.

**Conclusions:**

Many patients achieve long-term OS and LC without significant morbidity after salvage surgery and IORT, especially in case of clear margins.

## Background

The curative treatment of retroperitoneal and pelvic primary tumors often involves multidisciplinary approach. However, the local failure (LF) rates for various types of tumors range from 20 to 77% [1, 2], despite treatment regimens comprising surgery, radiotherapy, and/or chemotherapy. Such failures are associated with a worsening quality of life for the patient. Currently, salvage surgery is considered the only curative option for isolated recurrences of retroperitoneal and pelvic tumors (rRPT), especially in patients who have already undergone first-line radiotherapy; however, subsequent failures have been reported in more than 50% of such cases [3–5]. In other words, surgery alone cannot achieve satisfactory local control (LC). For such cases, adjuvant radiotherapy may reduce LF rates, especially in cases involving positive or close margins [3].

As noted above, treatment options for recurrent disease are limited for patients who have received first-line external beam radiotherapy (EBRT). However, intraoperative radiation therapy (IORT) can be used to administer single high doses of radiation to tumor beds to eliminate microscopic tumor foci while sparing the organs at risk. This modality also increases the likelihood that the tumor bed will be accurately identified without the restrictions of imaging exams [1, 5]. Furthermore, IORT can be used safely to administer additional doses of radiation to patients previously treated with EBRT [6], thus circumventing the dose limitation imposed by first-line treatment.

To date, few studies have evaluated the safety and efficacy of IORT for the treatment of rRPTs. Therefore, we aimed to evaluate the outcomes of surgery and IORT for rRPT at our institution in terms of LC and survival.

## Methods

### Patient and tumor characteristics

In this retrospective study, we analyzed the medical records of patients who underwent salvage surgery and IORT for isolated rRPTs between June 2004 and April 2015. Patients with metastatic disease or multiple recurrence foci were excluded.

The majority of cases (92.8%) were included in three different recurrent tumor groups: Colorectal Tumors, Retroperitoneal Sarcomas, or Gynecological Tumors. Although the patients also included one case each of a retroperitoneal recurrence of pancreatic tumor, pelvic recurrence of a primary soft tissue pelvic sarcoma, and pelvic recurrence of Ewing sarcoma. The cases were also subdivided in two histology: epithelial and sarcoma.

The characteristics of the 41 rRPTs patients who met the above-described criteria and were included in the study are listed in Table 1.

**Table 1:** Patient characteristics

	*N* = 41	%
Sex		
Female	27	65.8%
Male	14	34.2%
Age (years)		
Median	51 (range: 18–82)	
Groups stratified by tumor and histology		
Gynecologic	15	36.5%
-Uterine endometrioid adenocarcinoma	(4)	
-Uterine carcinosarcoma	(2)	
-Cervical adenocarcinoma	(4)	
-Cervical squamous cell	(4)	
-Ovarian adenocarcinoma	(1)	
Colorectal	12	29.5%
-Rectal adenocarcinoma	(8)	
-Colon adenocarcinoma	(3)	
-Anal squamous cell carcinoma	(1)	
Retroperitoneal sarcoma	11	26.8%
-Undifferentiated pleomorphic sarcoma	(6)	
-Liposarcoma	(4)	
-Fibrosarcoma	(1)	
Pelvic soft tissue sarcoma	1	2.4%
-Liposarcoma myxoid	(1)	
Ewing sarcoma	1	2.4%
-Ewing sarcoma	(1)	
Pancreas	1	2.4%
-Adenocarcinoma	(1)	
Tumor histology		
Epithelial	26	63.4%
Sarcoma	15	36.6%

### Treatment characteristics

Patients were initially treated via surgery combined with adjuvant or neoadjuvant therapies as appropriate for individual clinical cases. All recurrent cases were discussed in tumor board sessions that comprised the specialists involved with the treatment. When possible and indicated, patients underwent salvage surgery with IORT alone or in combination with EBRT.

**Fig. 1:**
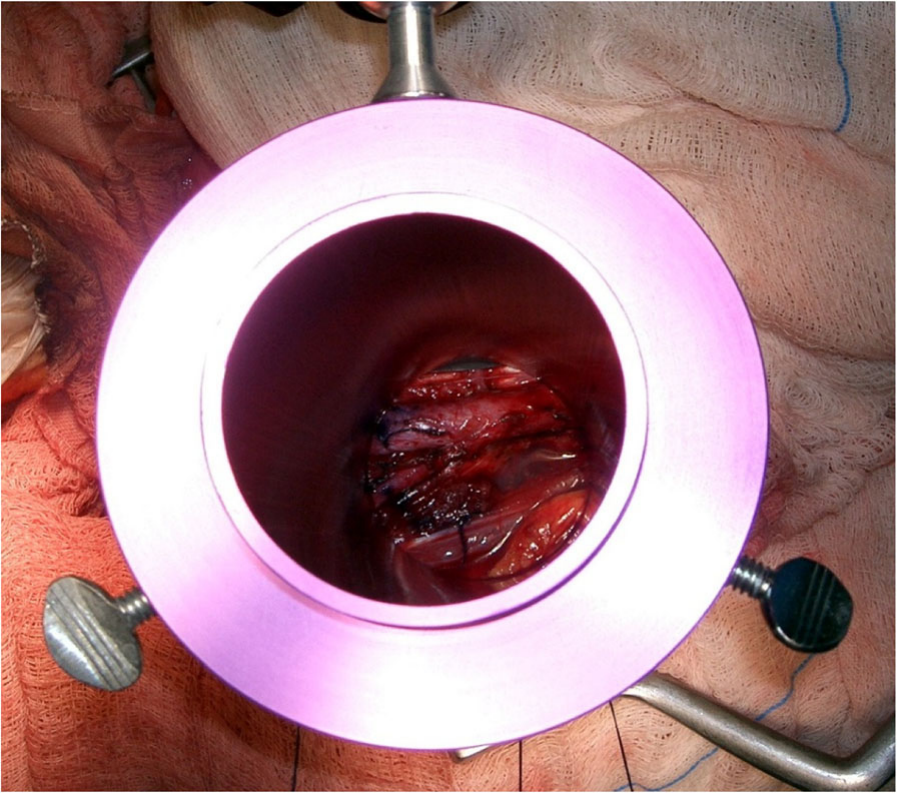
Delineation of the tumor bed in the pelvic region prior to IOERT

IORT was administered via either an electron beam from a Linac linear accelerator, which was directed by cylindrical applicators attached to the collimator, or a high-dose-rate (HDR) brachytherapy source. During surgery, the surgeon and radiation oncologist defined the target volume to be treated (Fig. 1). The anesthetized patients were transported to the Linac/HDR treatment room through an isolated 20-m route. Both the route and treatment room had been subjected to previous terminal cleaning. Cylindrical applicators with variable diameters of 6–12 cm and electron beams of 6–15 MeV or an Iridium 192 HDR source with HAM (Harrison–Anderson–Mick) applicator were used to ensure coverage of the tumor bed with margins of 10 mm. The characteristics of the various treatments are shown in Table 2.

**Table 2:** Treatment characteristics

	*N* = 44	%
IORT indication		
Pelvic recurrence	22	50%
Retroperitoneal recurrence	22	50%
Resection		
R0	36	88%
R1	08	12%
IORT device		
Electron (IOERT)	43	97%
HDR	01	3%
IOERT dose		
Median (first-line EBRT)	15 Gy (range:10–21 Gy)	
Median (no first-line EBRT)	12 Gy (range: 9–15 Gy)	
IOERT energy		
Median	9 MeV (range: 6–15 MeV)	
Association with adjuvant ebrt		
Yes	17	38.6%
No	27	61.4%
Association with chemotherapy		
Yes	11	25%
No	33	75%

Legend: *EBRT* External beam radiation therapy, *IOERT* Intraoperative electron radiation therapy

Outcomes of salvage surgical procedures were classified as follows: R0, free surgical margins; R1, resection with focally microscopically involved margins; and R2, visible or palpable residual tumor.

Forty-four treatments were delivered; these included 3 patients who each developed 2 recurrences at different anatomical sites. Only one treatment was performed using HDR, and this case was included in the colorectal tumor group; further, a 12-Gy radiation dose was prescribed to a 5-mm depth from the applicator surface and was associated with non-prior or adjuvant EBRT treatment. Fourteen cases (31.8%) involved recurrence sites that had been subjected to anterior EBRT at a median dose of 50 Gy (range: 45–60 Gy). Of these, 8 cases involved rectal tumors that received neoadjuvant treatment at a total dose of 45 Gy in fractions of 1.8 Gy; the first PTV encompassed the tumor and draining lymphatics and received a dose of 45 Gy, whereas the second PTV encompassed the tumor with a 2-cm safety margin in all directions and received a total dose of 50.4 Gy. In four cases involving uterine cervical tumors, the PTV encompassed the tumor bed and draining lymphatics and received an adjuvant dose of 45 Gy in fractions of 1.8 Gy. One case received adjuvant radiation therapy for the treatment of Ewing’s sarcoma; a total dose of 55.8 Gy in fractions of 1.8 Gy was delivered to a PTV encompassing the tumor bed with a 2-cm margin. The remaining case involved a pelvic sarcoma and received adjuvant treatment at a total dose of 60 Gy in 2-Gy fractions to a PTV that encompassed the tumor bed with 4-cm margins.

Another 13 cases (29.5%) had tumor beds containing a large volume of small intestine and had a high risk of developing actinic enteritis as a complication of EBRT. For these 27 cases, salvage treatment involved surgery and isolated IORT at a median dose of 15 Gy (range: 10–21 Gy). For the other 17 cases (38.6%), salvage treatments involved a combination of IORT at a median dose of 12 Gy (range: 9–15 Gy) and an additional adjuvant EBRT course at a total dose of 45 Gy in 25 daily fractions of 1.8 Gy to tumor bed plus 2 to 4 cm margins in all directions. One patient developed tumor recurrence in the para-aortic lymph node region and received an additional adjuvant course of EBRT to a PTV that encompassed the draining lymphatics in this region. All EBRT treatments realized before 2007 were treated with conformational-technique. From 2007 onwards the treatments were performed using intensity modulated radiotherapy (IMRT). Eleven treatments (25%) were administered in association with adjuvant (*n* = 7) or neoadjuvant (*n* = 4) chemotherapy.

### Patterns of failure

An LF was defined as recurrence or tumor progression within the IORT field. Regional failure (RF) was defined as recurrence or tumor progression in the retroperitoneal or pelvic region outside of the IORT field. Any other failure was defined as a distant failure (DF). Follow-up evaluations included physical examination, tumor markers, chemistry profiles and IORT anatomical site imaging with CT or MRI. Other imaging tests were ordered depending on patients’ clinical complaints. These were scheduled 30 days after IORT and every 3 months until 2 years, every 6 months for 5 years and annually thereafter.

### Study endpoints and statistical analysis

The study endpoints of LF, RF, DF, and overall survival (OS) were evaluated. LF was calculated from the date of IORT to the date of the first in-IORT field recurrence regardless of any previous DF, RF was measured from the date of IORT to the date of first outside-IORT field recurrence within the anatomical site(pelvic or retroperitoneal) even in the setting of local recurrence, DF was measured from the date of IORT to the date of first recurrence outside pelvic or retroperitoneal site. OS was calculated according to the Kaplan-Meier method from the date of IORT to the date of death or last contact. Differences in survival outcomes were compared using the log-rank test. The statistical analysis was performed using SPSS, version 24 (IBM, Inc., Armonk, NY, USA). A *p* value of ≤0.05 was considered statistically significant. Hazard ratio (HR) was used as a summary statistic of censored outcomes. The univariate and multivariate analyses were performed using the Cox regression method to identify risk factors.

Acute and chronic toxicities attributable to IORT were scored according to the RTOG/EORTC Radiation Toxicity Grading System [7] and the National Institutes of Health (NIH) Numeric Rating Scale (NRS-11) [8]. Toxicities attributed to the surgical procedure have been separately described.

## Results

Patients were followed for a median of 8.1 years (range: 4.4–11.7 years).

### Local control

**Fig. 2:**
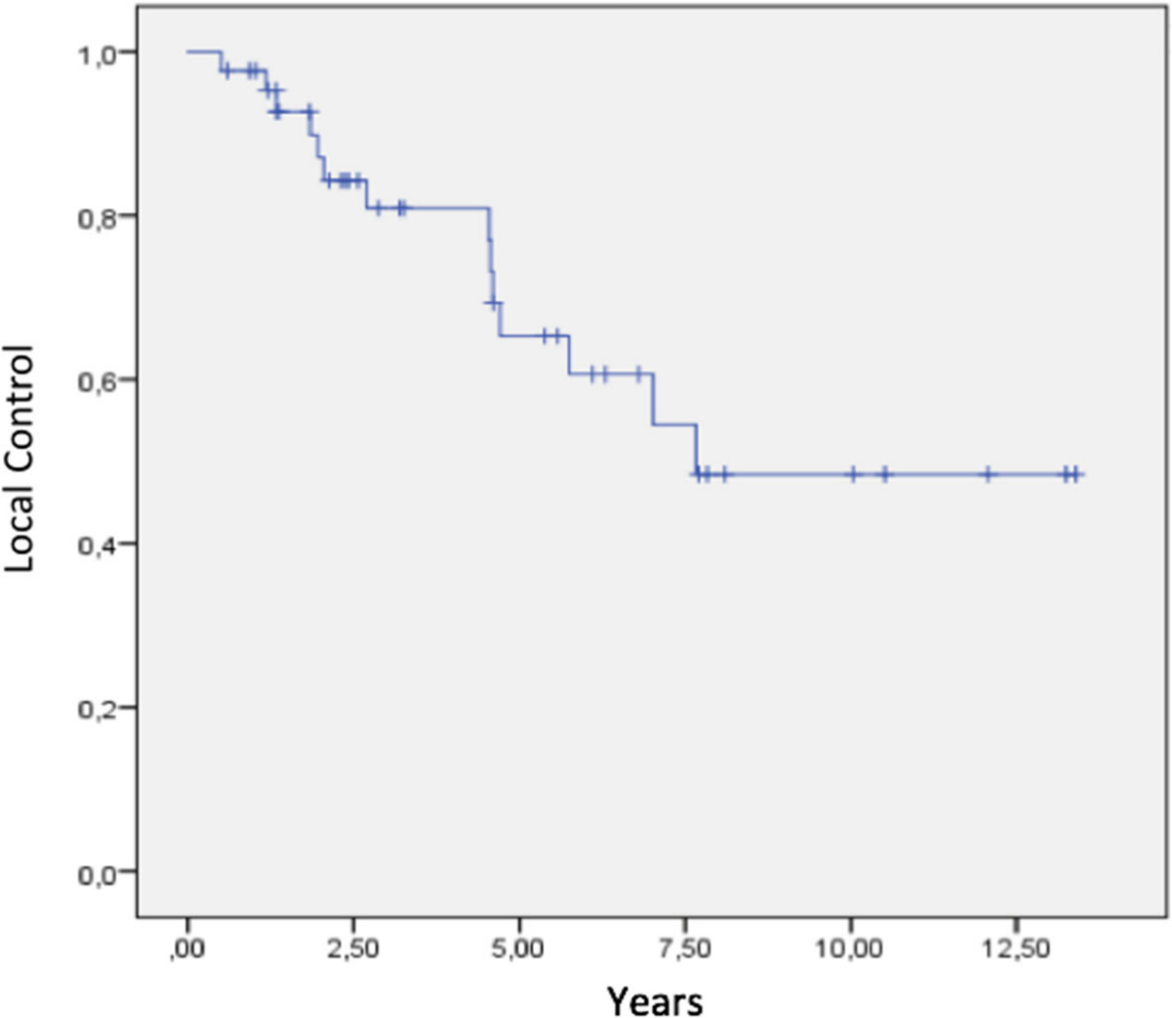
Local control for the entire cohort

**Fig. 3:**
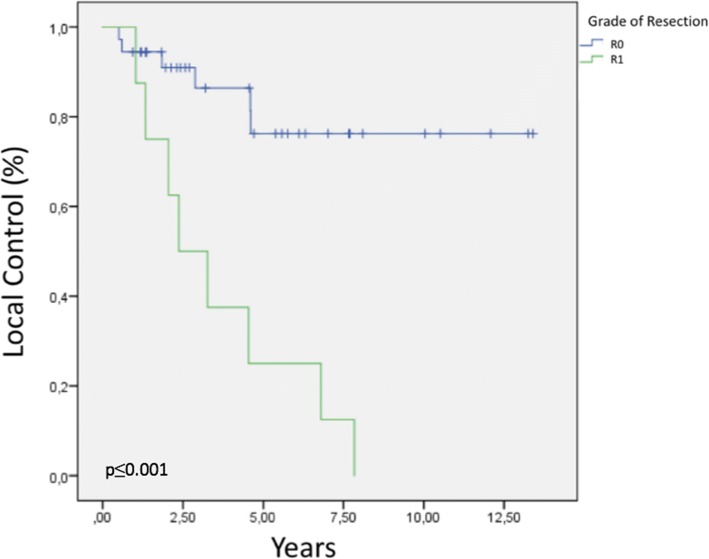
Local control by resection status

The 2, 5, and 8 year LC rates were 87.9, 64, and 49.8%, respectively. Figure 2 presents the Kaplan–Meier curve of LC for the entire cohort. In a subgroup analysis, the respective 2, 5, and 8 year LC rates were 90, 76, and 76% for R0 resection vs. 75, 25, and 0% for R1 resection, a significant difference (*p* ≤ 0.001). In summary, the achievement of R0 resection was associated with better LC (Fig. 3). A complete R0 resection was achieved in 36 treatments (81.8%), and an R1 resection was achieved in 8 cases (18.2%). No procedure yielded an R2 resection.

**Fig. 4:**
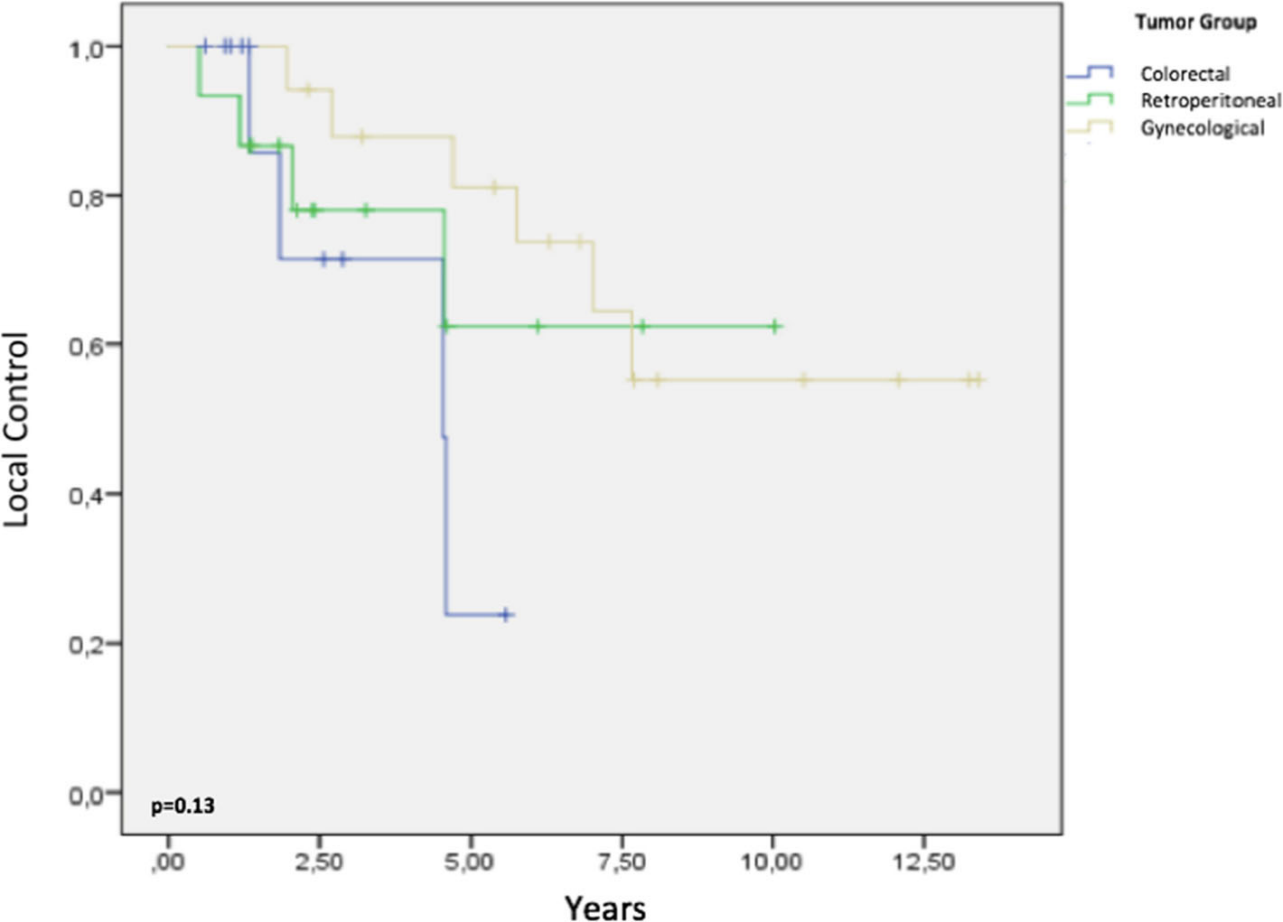
Local control by tumor group

The 2-, 5-, and 8-year LC rates for colorectal tumors were 71, 23, and 0%, respectively. Among patients with retroperitoneal sarcoma, the 2-, 5-, and 8-year LC rates were 86, 62, and 62%, respectively. The corresponding LC rates for gynecological tumors were 94, 81, and 55%, respectively (Fig. 4). In an analysis stratified according to histology subtypes, the respective 2-, 5-, and 8-year LC rates were 90, 70, and 47% for epithelial tumors and 80, 58, and 58% for sarcomas, respectively (*p* = 0.56).

Treatment-related variables and their effects on the 8-year LC rate are listed under Table 3.

**Table 3:** Associations of treatment-related variables with local failure (log-rank)

Treatment-Related Variables	Category	Local Control (8y)	*P*
Resection	R0	76.2%	< 0.001
	R1	0%	
EBRT (first treatment)	Yes	25.9%	0.092
	No	59%	
Association with adjuvant EBRT	Yes	46.9%	0.786
	No	54.4%	
Association with neoadjuvant chemotherapy	Yes	75%	0.854
	No	49.5%	
Association with adjuvant chemotherapy	Yes	62.5%	0.950
	No	45.9%	
Histology	Sarcoma	58%	0.56
	Epithelial	47%	
Tumor groups	Colorectal Tumors	23% (5y), 0% (8y)	0.06
	Retroperitoneal Sarcomas	62%	0.87
	Gynecological Tumors	55%	0.15

Legend: *Treat*. Treatment, *EBRT* External beam radiation therapy

In univariate analysis, only R1 resection significantly correlated with a high LF rate (HR: 6.7,95% CI: 2.3–19.7, *p <* 0.001). In multivariate analysis of the three tumor groups (colorectal, retroperitoneal, and gynecological), a recurrent gynecological tumor was associated with better LC, although this association was only marginally significant (HR: 0.26,95% CI: 0.06–1.04, *p* = 0.058).

### Overall survival

**Fig. 5:**
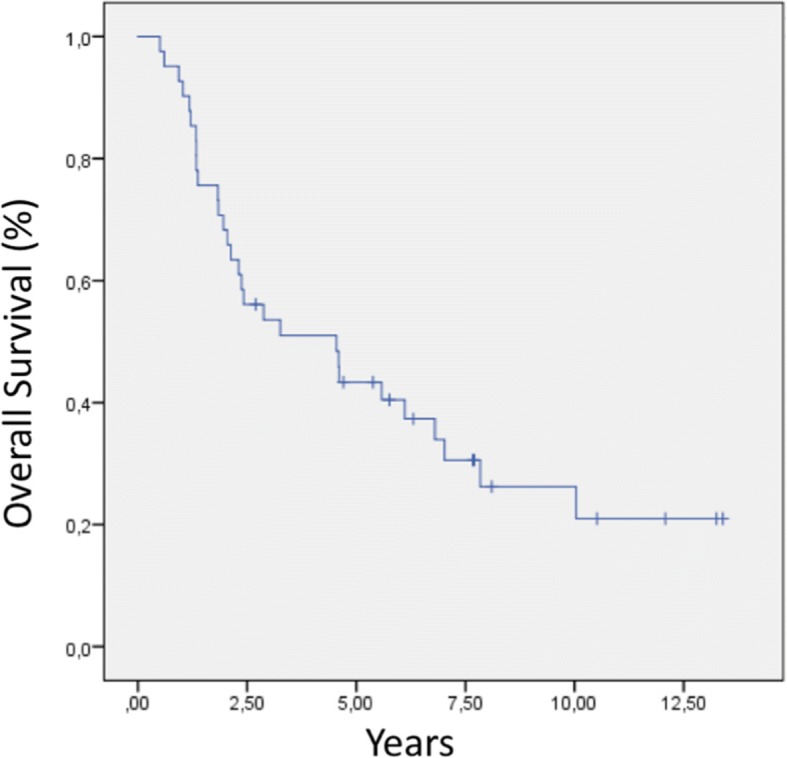
Overall survival for the entire cohort

**Fig. 6:**
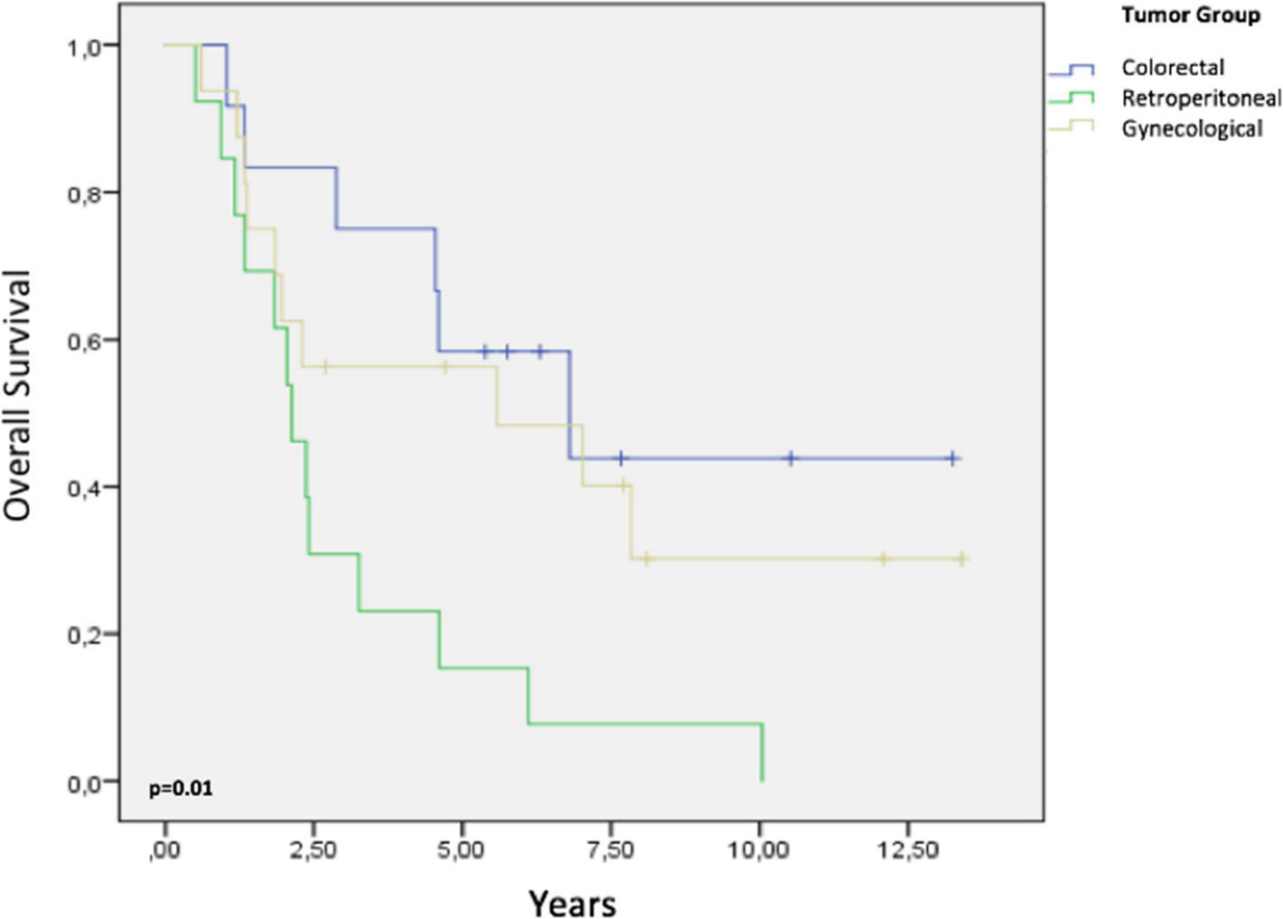
Survival analysis by tumor group

Among all patients, the 2-, 5-, and 8-year OS rates were 68, 43, and 26%, respectively. The Kaplan–Meier curve of OS for the entire cohort is presented in Fig. 5. Regarding the three tumor groups, the respective 2-, 5-, and 8-year OS rates were 83, 58, and 43% for colorectal tumors (*p* = 0.09); 61, 15, and 7% for retroperitoneal sarcomas (*p* = 0.005); and 62, 56, and 30% for gynecological tumors (*p* = 0.43), respectively (Fig. 6). Regarding histology subtypes, the respective 2-, 5-, and 8-year OS rates were 73, 57, and 40% for epithelial tumors and 60, 20, and 6% for sarcomas, respectively (*p* = 0.005).

**Fig. 7:**
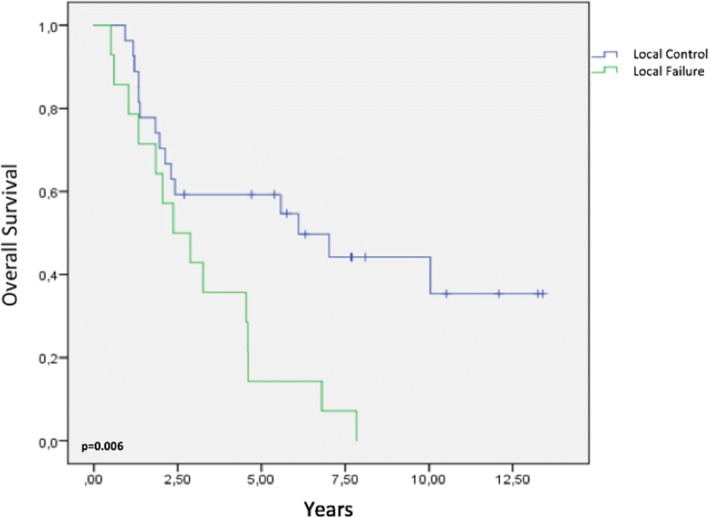
Survival analysis by local control status

Patients with LF had a significantly worse OS compared with patients with LC (5-year OS: 14% vs. 59%, *p* = 0.006) (Fig. 7). Six patients (14.6%) experienced RF and 24 (58.5%) experienced DF. Patients with and without DF had 5-year OS rates of 36 and 52%, respectively (*p* = 0.042).

The disease-specific mortality rates were 32, 56, and 71% at 2, 5, and 8 years, respectively. The treatment-related variables and their effects on OS are presented in Table 4.

**Table 4:** Associations of treatment-related variables with overall survival after 8 years (log-rank)

Treatment-Related Variables	Category	Overall Survival	*P*
Sex	Male	28.6%	0.876
	Female	25.5%	
Age (years)	< 51	30.6%	0.243
	> 51	23.5%	
Region of recurrence	Pelvic	20.8%	0.588
	Retroperitoneal	33.4%	
Local failure	Yes	44.2%	0.006
	No	0%	
Distant failure	Yes	10.3%	0.042
	No	52.9%	
Resection grade	R0	35.8%	0.12
	R1	0%	
EBRT (first-line treatment)	Yes	17.9%	0.109
	No	30.4%	
Association with adjuvant EBRT	Yes	21.4%	0.687
	No	34.7%	
Association with neoadjuvant chemotherapy	Yes	0%	0.157
	No	28.6%	
Association with adjuvant chemotherapy	Yes	38.1%	0.347
	No	23.1%	
Histology	Sarcoma	6,7%	0.005
	Epithelial	40%	
Group of tumors	Colorectal Tumors	43%	0.09
	Retroperitoneal Sarcomas	7%	0.005
	Gynecological Tumors	30%	0.43

Legend: *EBRT* External beam radiation therapy

In a univariate analysis of the entire cohort, the following prognostic factors were found to correlate with the mortality rate: LF (HR: 2.7,95% CI: 1.31–5.95, *p* = 0,006), absence of DF (HR: 0.43,95% CI: 0.18–0.99, *p* = 0.042), recurrent retroperitoneal sarcoma (HR: 2.81,95% CI 1.33–5.94, *p =* 0.007), and sarcoma histology (HR: 2.75,95% CI 1.31–5.75, *p =* 0.007).

The results of the univariate analysis are presented in Table 5.

**Table 5:** Results of a univariate analysis of the associations of various clinical characteristics with overall survival

Clinical Characteristic	Category	HR (Univariate) 95% CI
Sex	Male	1.06 (0.48–2.36)
	Female (Ref.)	
Age (≥ vs. < 51 years)	< 51 years	1.57 (0.72–3.42)
	≥51 years (Ref.)	
Region of recurrence	Pelvic	0.81 (0.38–1.70)
	Retroperitoneal (Ref.)	
Local failure	No	2.79 (1.31–5.95)
	Yes (Ref.)	
Resection grade	R0	1.87 (0.82–4.27)
	R1 (Ref.)	
Association with first-line EBRT	Yes	0.53 (0.24–1.16)
	No (Ref.)	
Association with adjuvant EBRT	Yes	1.16 (0.55–2.45)
	No (Ref.)	
Association with neoadjuvant chemotherapy	Yes	0.42 (0.12–1.43)
	No (Ref.)	
Association with adjuvant chemotherapy	Yes	1.65 (0.57–4.78)
	No (Ref.)	
Distant failure	Yes	0.43 (0.18–0.99)
	No (Ref.)	
Histology	Sarcoma (Ref.)	2.75 (1.31–5.75)
	Epithelial	
Colorectal Tumor	Yes (Ref.)	0.47 (0.19–1.16)
	No	
Retroperitoneal Sarcoma	Yes (Ref.)	2.81 (1.33–5.94)
	No	
Gynecological Tumor	Yes (Ref.)	0.73 (0.34–1.58)
	No	

In a multivariate analysis the presence of LF (HR: 2.71, 95% CI: 1.24–5.92, *p =* 0.012) and retroperitoneal recurrent sarcoma were found to influence the survival rate (HR: 3.40, 95% CI: 1.27–9.02, *p* = 0.014).

### Morbidity

Sixteen patients experienced toxicities attributable to the surgical procedure, including 3 cases of intestinal obstruction, 3 of lymphocele, 2 of persistent lower limb edema, 2 of ureter stenosis, 2 of operative wound dehiscence, and 1 each of perineal fistula, enteric fistula, pancreatic fistula, and retroperitoneal hematoma. Surgery was required to treat the cases with intestinal obstruction, ureter stenosis, enteric fistula, and operative wound dehiscence. No deaths due to surgical complications were reported. Additionally, 13 patients (31%) developed complications related to IORT. Seven patients (17%) developed acute pain with a severity of 4–6 on a 10-point scale that was associated with the procedure and could be controlled with opioids. Two patients (5%) developed acute grade 2 lower GI enteritis. Chronic toxicity was observed in 3 (7%) patients with chronic neuralgia controlled with opioids and antidepressants. 1 (2,5%) patient developed ischiatic osteomyelitis 1 year after IORT. No deaths were attributed to toxicities.

## Discussion

This report describes our single-center experience with a multimodality approach comprising salvage surgery and IORT for the treatment of rRPTs. Notably, our analysis demonstrated satisfactory long-term LC rates, particularly for cases in which an R0 resection was achieved.

Our study included several histological tumor types and thus demonstrated the different clinical scenarios wherein IORT can be employed. In a study involving 128 patients, Cambeiro et al. [9] also analyzed the results of IORT for the treatment of several types of tumors, such as soft tissue sarcomas as well as head and neck, uterine, and colorectal tumors. The authors used a treatment regimen similar to that used in our study, with association of EBRT (median dose 46 Gy) and IORT (median dose 15 Gy) in 58% of the cases. In a multivariate analysis, only the degree of resection statistically significantly influenced LC, such that the patients who underwent R2 resection had a 2.2-fold higher risk of treatment failure (95% CI: 1.2–4.1; *p* = 0.007). In that study, patients with histologically epithelial tumors had lower survival rates. By contrast, in our study, we observed worse survival outcomes associated with sarcoma histology, which we attribute to the presence of this histology in tumors with more aggressive clinical behavior, such as recurrent retroperitoneal and uterine sarcomas. In relation to dose intensity, the authors concluded that the treatments performed with a combination of IOERT and EBRT, with a median value for tumor control standardized to 2 Gy equivalents (EQD2) ≥ 62 Gy had a statistically significant influence on overall survival, compared to treatments with isolated IOERT, with a median value of EQD2 of 31.2 Gy (HR 2.2, 95% CI: 1.1–4.1). In our study, the median value of the EQD2 for the treatments performed with the association of the two modalities and with isolated IORT were 66.2 Gy and 31.2 Gy respectively, but we did not verify influence on overall survival, probably due to the restricted number of patients and by the limitations of a retrospective study.

Below, we further discuss the outcomes of our study in terms of the type of recurrence.

### Recurrent colorectal tumors

Recurrent colorectal cancers present unique challenges in terms of management, as previous EBRT limits the available options for further radiation treatment. However, IORT may be indicated in select cases.

Previous studies of IORT have reported variable LC and OS outcomes. One extensive IORT review demonstrated a significant improvement in LC among patients with locally recurrent pelvic tumors [10]. Furthermore, in a meta-analysis of > 3000 patients, Mirnezami et al. observed benefits with IORT in terms of LC, disease-free survival, and OS among patients with recurrent rectal and advanced colorectal cancers [11]. A Cleveland Clinic study reported a 1 year LF rate of 16% and 3 year OS rate 49% among patients who underwent IORT for locally advanced and recurrent rectal cancers [12], and Roeder et al. reported 5 year LF and OS rates of 59 and 30%, respectively, in a similar clinical situation [3]. Hyngstrom et al. reported 1, 3, and 5 year LF rates of 16, 40, and 44%, respectively, with high-dose-rate intraoperative brachytherapy [13].

In one case involving a recurrent colorectal tumor in the lateral pelvic wall, we performed intraoperative brachytherapy using a HAM applicator because the anatomical tumor bed location did not allow adequate electron-beam therapy.

Several previous studies have identified surgical resection margin status as an important factor affecting LC during treatment of patients with local recurrences of colorectal cancer. In a recent clinical review, Haddock MG [14] extensively analyzed several studies and demonstrated that in selected series involving IORT for the treatment of recurrent rectal cancer, the LC rates were 60–80% and the 5-year survival rates were 40–50%. Among cases with microscopically positive margins, the LC rates ranged from 30 to 60% and the 5-year survival rates ranged from 20 to 30%. Other studies have also evaluated the influence of resection grade on the LC of recurrent colorectal tumors. For example, in a study by Roeder et al., patients with a status of R0 after the resection of locally recurrent rectal cancer were found to have 5 year LC and OS rates that were respectively threefold and fivefold better than those of patients with incomplete resections [3]. Consistent with that report, three resections of recurrent rectal tumors (25%) in our series achieved an R1 status, and none obtained LC. Holman et al. reported a 5 year LF of 45% in a pooled analysis of 565 patients treated with IORT for advanced and recurrent rectal cancers and identified the resection grade as a risk factor for LC [15]. Dresen et al. [16] also observed worse LC rates following R1 resection in an analysis of 147 patients with local recurrences of rectal cancer. In his series, the median OS duration was 28 months, whereas the 5-year OS, DFS, metastasis-free survival, and LC rates were 31.5, 34.1, 49.5, and 54.1%, respectively. Resections R0, R1 and R2 was achieved in 84 (57.2%), 34 (23.1%), and 29 (19.7%) patients respectively. For patients with a resection R0, median Os was 59 months and 5-years OS and LC were 48,4% and 68,9% respectively (*p* < 0,001). In our study, these treatments yielded 9 R0 (75%) and 3 R1 resections (25%), with a 5 year LC rate of 73%. Consistent with that report results, none of the resections with R1 status obtained local control.

Despite the favorable LC and OS outcomes of IORT, treatment complications are reported with relative frequency in the literature. In our experience, we observed chronic neuralgia in 3 patients and osteomyelitis in 1 patient. Suzuki et al. reported a 5 year LC rate of 60% among patients who received EBRT before or after adjuvant IORT [17]; however, this outcome was unfortunately accompanied by a grade 3 toxicity incidence exceeding 30%. Similarly, Willet et al. reported a complication rate of 30% after preoperative EBRT associated with IORT for recurrent pelvic tumors [18]; here, most events involved soft tissue or sacral injury and pelvic neuropathy. Roeder et al. identified wound healing disturbance as the most common complication (20% of patients), followed by abscess or fistula formation (16%) and severe chronic pain (8%) [3].

Finally, most relevant literature reports have reported DF with this type of neoplasia. Consistent with those reports, half of the 12 cases of recurrent colorectal tumors in our study developed a DF, including 6 cases of liver metastasis.

### Recurrent retroperitoneal sarcomas

Approximately 38% of all sarcomas arise in the retroperitoneum [19], and surgery is currently the main treatment with curative intent. In contrast to sarcomas of the extremities, however, wide surgical margins are often not achievable in the retroperitoneum; accordingly, local progression is the dominant pattern of failure. As the retroperitoneum contains many critical organs with low radiation tolerances, adequate dose delivery via EBRT either cannot be achieved or would result in excessive toxicity. By contrast, IORT facilitates the delivery of a single high radiation dose to the tumor bed during surgery while sparing the surrounding organs at risk via physical distance from the radiation field or adequate lead shielding. The total combined dose from IORT plus moderate EBRT increases the possibility of LC while reducing toxicity.

Regional failure is commonly observed among patients with retroperitoneal sarcoma, including 5 patients in our study; an additional 5 patients experienced DF.

No randomized data regarding radiotherapy for retroperitoneal sarcoma are currently available. An ongoing EORTC study (NCT01344018), Surgery with or without Radiation Therapy in Untreated Nonmetastatic Retroperitoneal Sarcoma (STRASS), is currently recruiting participants. Furthermore, propensity score-matched analyses of more than 9000 patients with resected retroperitoneal sarcoma with or without preoperative or postoperative irradiation in the National Cancer Data Base demonstrated improved median OS in the irradiation group compared with the no-irradiation group, regardless of the irradiation time (110 months for preoperative vs. 89 months for postoperative vs. 66 months for no irradiation) [20].

Regarding LC, other authors have reported results similar to ours. Petersen et al. evaluated the management of 44 cases of recurrent retroperitoneal soft tissue sarcoma treated with IORT at the Mayo Clinic; the reported LF rate of 39% was comparable with our series. The authors also reported a 5 year OS rate of 48%, with no differences were found by primary or recurrent tumor status [21].

Hager et al. compared two groups of patients; although all underwent surgery, half also received radiation therapy [22]. As in our study, Hager and colleagues typically administered a median IORT dose of 15 Gy using an electron energy of 6 MeV. The combination of surgery and IORT significantly improved survival outcomes, compared with surgery alone (*p* = 0.04). For all patients, resections R1 and R2 resection have a decreasing in 5 years survival rate by 7.6% and by 34.7% respectively compared to R0 resection.

Recently, Roeder et al. [23] published a retrospective study involving 156 patients among whom 87 had recurrent tumors. Total 114 patients were treated with a combination of IORT (median dose: 15 Gy) and EBRT (median dose: 45 Gy). During a median follow-up of 38 months, the LC rates at 3 and 5 years were 57 and 50%, respectively. In a multivariate analysis, the tumor grade resection margins and the association with EBRT remained statistically significant.

### Recurrent gynecological tumors

Patients with recurrent gynecological tumors (e.g., tumors of the uterine cervix, endometrium, and ovary) often present with lesions on the pelvic walls and/or involvement of the pelvic or paraaortic lymph nodes. Again, a previous treatment history of high-dose EBRT for these tumors limits the options for salvage radiation therapy. Accordingly, IORT is an important therapeutic option.

IORT has been studied for the treatment of recurrent gynecologic tumors since the 1990s. In a Mayo Clinic study of 148 patients, 125 patients had recurrent gynecological tumors and 113 received IORT associated with EBRT [24]. In that study, the 5 year LF rate was 40% and the 5 years OS was 27%. Furthermore, R2 resection was associated with a worse 5 year OS rate, compared with R0 or R1 resection (31% vs 13%, *p* = 0.01).

Tumors of the uterine cervix are common in Brazil and are often diagnosed at advanced stages. Some retrospective studies in the literature have evaluated the use of IORT for the treatment of recurrent uterine cervical cancer. Tran et al. evaluated 17 patients treated with orthovoltage IORT at a median dose of 11.5 Gy [25]. In that study, the LC, metastasis-free survival, and specific survival rates were 45, 60, and 46%, respectively. Similar to our treatment scheme, a Spanish study reported the outcomes of 36 patients with recurrent primary cervical tumors who were treated with IORT (median dose: 15 Gy) alone or combined with EBRT (45 Gy at 1.8 Gy per fraction) if prior radiotherapy had not been administered [26]. In that study, the 10 year LC was 47%, and factors that adversely affected LC included parametrial margin involvement, R1 resection, and pelvic lymph node involvement.

The Mayo Clinic reported the outcomes of 25 patients treated with IORT for recurrent endometrial cancer [27]. Most patients presented with involvement of the pelvic sidewall or paraaortic nodes. In that study, 21 of 25 patients received EBRT (median dose: 45 Gy). The median IORT dose of 15 Gy was consistent with our study. The median survival duration was 57 months, and the 5 year OS rate was 47%. The authors of that study reported that the resection grade influenced survival, with 5 year OS rates of 71, 40, and 0% among R0, R1, and R2 cases, respectively. LF within the IORT field and DF were observed in 4 (16%) and 6 patients (24%), respectively. Given the small number of patients with recurrent gynecological tumors in our study, we could not analyze the influence of histological type on survival. However, other studies have reported better survival outcomes for recurrent endometrial tumors treated with IORT. In a study involving 36 patients with recurrent gynecological malignancies conducted by Arias et al. [28], an endometrial histology was found to correlate with better rates of local PFS (*p* = 0.017) and OS (*p* = 0.038). Furthermore, older patients exhibited significantly better distant PFS outcomes (*p* = 0.015), and patients with endometrial cancer tended to better distant PFS relative to patients with recurrent cervical and vulvar tumors.

Investigators at Stanford University reported the use of orthovoltage IORT to treat 22 patients with recurrent ovarian cancer [29]. In that study, a median dose of 12 Gy was administered to various sites, including the pelvis, paraaortic nodes, inguinal nodes, and porta hepatis. Nine, 5, and 6 patients received whole-abdominal EBRT, loco-regional EBRT, and associated chemotherapy, respectively. The median survival duration was 26 months, and the 5 year OS and disease-free survival rates were 22 and 18%, respectively. LC and RC was 68% at 22 months. Furthermore, 55% of patients experienced DF; consistent with our study in which 9 patients (60%) experienced DF, the authors identified this failure pattern as an important influence on OS.

For the various types of tumors, we observed a direct influence of the surgical margin status on LC. Some authors believe that R1 or R2 resection may be a consequence of selection of tumor variants with more aggressive biological behaviors following the initial course of radiotherapy and chemotherapy [16]. Despite progress in the quality and precision of imaging exams and the rigorous selection of clinical cases, a true evaluation of the possibility of obtaining a R0 surgical margin may only be possible during surgery. In our experience, we considered cases with increased risk and those with tumors very close to bone structures, such as recurrences of pelvic tumors in the pre-sacral space, recurrences of retroperitoneal sarcomas in the paravertebral region, and lesions adhered to vascular structures. Such cases may be considered “borderline” and should be the subject of a more extensive evaluation to determine the indications and contraindications for IORT. The intraoperative identification of R1 or R2 margins might indicate the need for a dose increase, which is often difficult to accommodate depending on the previously administered EBRT dose and the presence of high-risk anatomical structures in the tumor bed. Other alternative treatment techniques for recurrences of pelvic tumors have been reported in the literature. Murray et al. [30] analyzed 17 studies of stereotactic ablative radiotherapy (SART) for the treatment of 205 patients with recurrent malignant disease within the pelvis. SART has the advantage of allowing the previous treatment planning, optimizing the coverage of the target volume of treatment. The treatment being performed in a fractionated dose regimen allows the radiobiological advantage of better protection of normal structures.

The authors reported 1-year CL indices ranging from 51 to 100% and a low rate of grade 3 or 4 complications.

### Morbidity

Surgeries performed for the salvage treatment of pelvic or retroperitoneal tumor recurrences are extensive, debilitating, and potentially complicated by previous treatments. First-line EBRT induces fibrosis in tissues surrounding the recurrent tumor and increases the difficulty of the surgical procedure. We observed some serious complications; however, they were fortunately not fatal. Of these complications, 70% occurred within 90 days of surgery and could be attributed to the surgical procedure.

Peripheral neuropathy is the most commonly reported toxicity attributed to pelvic IORT. IORT-related neuropathies usually present with pain but no significant motor or sensory loss. The pain is usually chronic, possibly severe, and is often manageable with analgesics, including opioids. In our study, this complication occurred in 7 patients and mainly affected those who received treatment for recurrent pelvic region tumors (5 cases). In such cases, pain was likely attributable to the presence of nerve structures in the treatment field (i.e., very near the tumor bed); here, nerves cannot be shielded and thus receive the full IORT dose.

Some authors have attempted to correlate the incidence of neuropathy with the IORT dose. A Mayo Clinic analysis involving 51 patients treated with IORT (doses: 10–25 Gy) in association with EBRT for primary or recurrent pelvic tumors observed grade 1–3 neuropathy in 32% of patients [31]. Haddock et al. analyzed the relationship between the IORT dose and incidence of neuropathy in patients receiving treatment for recurrent rectal tumors and noted that IORT doses of ≥12.5 Gy were associated with an incidence of 5% grade 2 or 3 neuropathy, whereas doses > 15 Gy were associated with 14% incidence of grade 2 or 3 neuropathy [32]. Of the 7 patients who developed pain in our study, 6 received an IORT dose ≥15 Gy (range: 15–20 Gy). However, given the small number of patients with this complication, it was not possible to establish a statistical association between the IORT dose and the incidence of neuropathy.

## Conclusions

In this study, a rigorous adherence to follow-up, including accurate imaging studies, facilitated the detection of isolated recurrent retroperitoneal and pelvic tumors. For selected cases, salvage treatment comprising surgery and IORT, either alone or with EBRT, yielded satisfactory LC and survival outcomes with acceptable morbidity. Despite this favorable LC outcome, however, survival was strongly influenced by the occurrence of DF. Our findings underscore the importance of individualized case discussions by tumor boards, as well as the need for additional studies to identify more effective and targeted systemic treatments for this specific group of patients.

## Abbreviations

CI: Confidence interval
DF: Distant failure
EBRT: External beam radiation therapy
HDR: High dose rate
HR: Hazard ratio
IOERT: Intraoperative electron radiation therapy
IORT: Intraoperative radiation therapy
LC: Local control
LF: Local failure
OS: Overall survival
PTV: Planning target volume
RF: Regional failure
